# Giant cell tumor-like lesion of the urinary bladder: a report of two cases and literature review; giant cell tumor or undifferentiated carcinoma?

**DOI:** 10.1186/1746-1596-4-48

**Published:** 2009-12-31

**Authors:** Kemal Behzatoğlu, Haydar Durak, Şule Canberk, Övgü Aydın, Gülben Erdem Huq, Meltem Oznur, Gül Özyalvaçlı, Pelin Yıldız

**Affiliations:** 1 Department of Pathology, Istanbul Education and Research Hospital, Istanbul, Turkey; 2 Department of Pathology, Cerrahpasa Medical Faculty, Istanbul, Turkey; 3 Department of Pathology, Istanbul Education and Research Hospital, Istanbul, Turkey

## Abstract

**Summary:**

Giant cell tumor, excluding its prototype in bone, is usually a benign but local aggressive neoplasm originating from tendon sheath or soft tissue. Malignant behavior is uncommon. Visceral organ involvement including urinary bladder is rare. Giant cell tumors in visceral organs usually accompany epithelial tumors and the clinical behavior of giant cell tumor in urinary bladder is similar to its bone counterpart. Here, we report two cases of giant cell tumor located in urinary bladder in comparison with nine reported cases in the English literature. Concurrent noninvasive urothelial carcinoma was also described in all these previous reports and only one patient with follow-up died of disease. One of the two cases we present had no concurrent urothelial tumor at the time of diagnosis but had a history of a low grade noninvasive urothelial carcinoma with three recurrences. The histology of these two cases was similar to the giant cell tumor of bone and composed of oval to spindle mononuclear cells with evenly spaced osteoclast-like giant cells. Immunohistochemically, the giant cells showed staining with osteoclastic markers including CD68, TRAP, and LCA. Immunohistochemical expression of vimentin, CD68, LCA, and smooth muscle actin in mononuclear cells supported a mesenchymal origin with histiocytic lineage. The histologic and immunohistochemical properties in our cases as well as their clinical courses were consistent with a giant cell tumor. Consequently, tumors in urinary bladder showing features of giant cell tumor of bone may also be considered and termed "giant cell tumor".

## Introduction

Giant cell tumor (GCT) usually originates from bone, tendon sheath or soft tissue and is composed of oval, plump mononuclear cells and osteoclasts with a slow rate of growth and low incidence of malignant behavior. Tumors with similar histology were described in a variety of visceral organs such as pancreas, ovary, larynx, urinary tract, thyroid and salivary glands [1-6]. The etiology and histogenesis of GCT are controversial and have largely remained unexplained. GCT is exceptionally rare in urinary bladder with only nine cases reported in English literature to date [7-12]. GCT is usually accompanied by an epithelial neoplasm in visceral organs including the urinary bladder.

Holtz *et al*. was the first to describe GCT in urinary bladder and since then it has been referred to by various names such as carcinosarcoma, GCT, giant cell reparative granuloma, osteoclast-like GCT, and osteoclast-rich undifferentiated carcinoma [7-10,12]. Recent reports suggest that tumors in visceral organs such as urinary bladder and pancreas should be classified in the undifferentiated carcinoma group considering the common accompaniment of epithelial neoplasms, as well as immunohistochemical expression of epithelial markers and p53, and aggressive course in some cases [12,13]. On the other hand, except from high grade or invasive neoplasms GCT-like lesions may also accompany to those with benign or non-invasive low grade histology or even arise *de novo*. In addition *de novo *tumors as well as some soft tissue tumors may also express epithelial markers immunohistochemically. These findings indicate that histogenesis of GCT is still controversial and that at least some lesions may be an overdiagnosed as "undifferentiated carcinoma".

In this study, we evaluated the clinical, histologic and immunohistochemical findings in two cases of urinary bladder tumors having morphological findings similar to GCT of bone, compared them with previous cases reported in English literature, and discussed the histogenesis and nomenclature of GCT.

## Case Reports

### Clinical Histories

Case 1. Cystoscopic examination of a 56 year old man presented with hematuria in March 2003 revealed a 1 cm mass at the posterior wall of urinary bladder. The pathologic diagnosis was noninvasive low grade urothelial carcinoma. Tumor recurrences were seen at cystoscopic controls in August 2003, March 2004 and August 2004. The histologic appearances of the recurrences were identical to the first tumor. In June 2005, the patient had an additional cystoscopic examination due to severe painful hematuria. An ulcerated mass located at lateral wall and trigone was resected transurethrally and sent for pathologic examination. No other lesions were detected by clinical and radiological examinations of the whole body. He has been free of disease for 43 months.

Case 2. The second case was a 74 year old man presented with severe hematuria. Cystoscopy was performed and biopsy was taken. The patient has been free of disease for 50 months.

## Materials and methods

The English literature was scanned and nine cases of GCT of urinary bladder were found. The primary diagnosis of our cases belongs to KB and HD, respectively. Clinical histories of the patients were obtained from clinical files. All hematoxylin-eosin stained slides were examined and the most representative slides were chosen for immunohistocemical examination. Immunohistochemistry was performed using the standardized streptavidin-biotin-immunoperoxidase complex with 3'3' diaminobenzidine as chromogen (large volume DAKO, Carpenteria, CA, USA) LSAP+ kit, according to the manufacturer's instructions. Specifications for the various immunostains that were used are listed in Table 1.

**Table 1:** List of Antibodies for Immunohistochemical Studies

Antibody	Clone	Dilution	Antigen retrieval method	Source
Vimentin	V9	1:150	Citrate buffer (20 min)	Neomarkers, Fremont, CA, USA

Epithelial membrane antigen (EMA)	GP1.1	1:150	Citrate buffer (20 min)	DBS, Pleasanton, CA

Pan-cytokeratin	AE1/AE3	1:50	Citrate buffer (20 min)	Neomarkers

Cytokeratin 7	OV-TL 12/30	1:200	Tripsin (10 min at 37°C)	Neomarkers

Cytokeratin 20	Ks20.8	1:100	Tripsin (10 min at 37°C)	Neomarkers

Desmin	D33	1:100	Citrate buffer (20 min)	Neomarkers

α-Actin (SMA)	1A4	1:200	Citrate buffer (20 min)	Neomarkers

S-100 protein	4C4.9	1:150	Citrate buffer (20 min)	Neomarkers

Leukocyte common antigen (LCA)	Cocktail PanLCA	1:200	Citrate buffer (20 min)	Neomarkers

CD68	PG-M1	1:100	Citrate buffer (20 min)	Neomarkers

Ki-67	SP6	1:200	Citrate buffer (20 min)	Neomarkers

P53	DO7	Prediluted	Citrate buffer (20 min)	Dako, Carpenteria, CA, USA

TRAP	26E5	Prediluted	Citrate buffer (20 min)	Neomarkers

## Results

### Pathologic Findings

**Figure 1:**
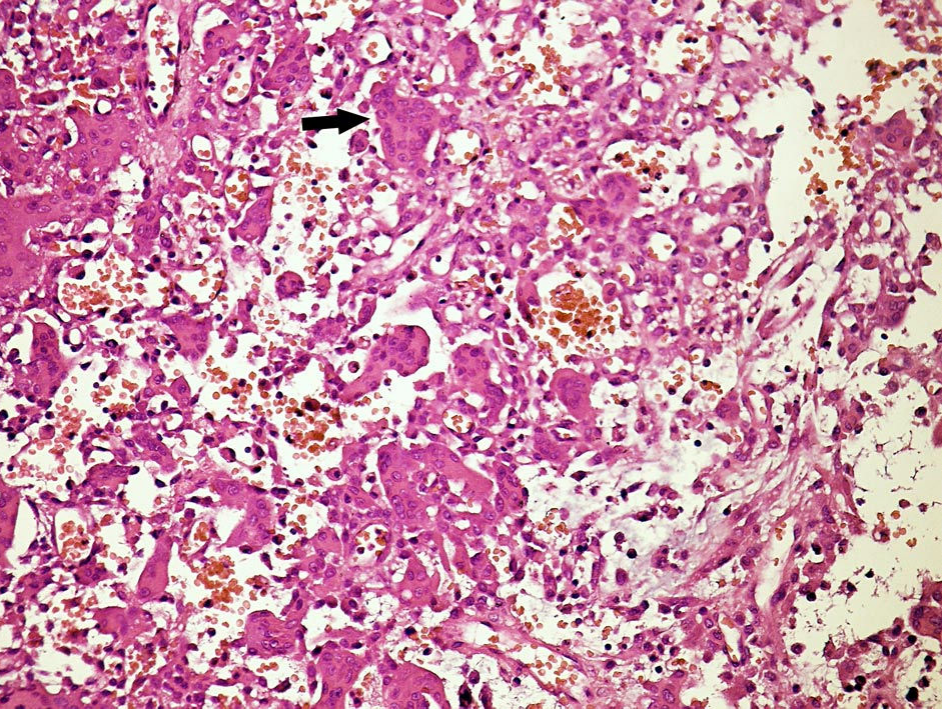
**Numerous osteoclasts (arrow) and few mononuclear cells between blood lakes in Case 2. (H&E, × 300)**.

**Figure 2:**
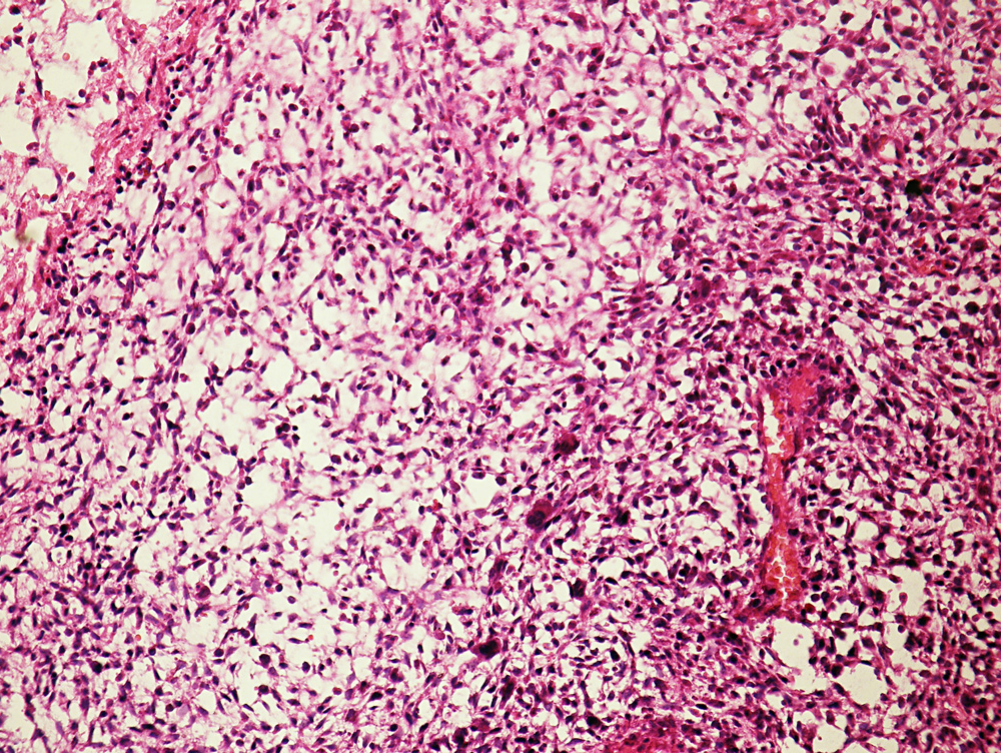
**Spindled mononuclear cells and dispersed osteoclasts in Case 1. (H&E, × 200)**.

At light microscopy, both tumors displayed the typical morphological properties of GCT of bone composed of mononuclear and multinucleated giant cells (osteoclasts) forming clusters and nodules. Mononuclear cells were oval, plump or spindle with scant eosinophilic cytoplasm (Figure 1, 2). Mitoses in mononuclear cells (1-2/10 HPF) were present only in Case 1. Cytological atypia and pleomorphism in mononuclear cells were minimal in both cases. Rich vascularized stroma with extravasated erythrocytes and lakes filled with blood were also noted. Giant cells morphologically similar to osteoclasts clustered mostly in hemorrhagic areas. Giant cells had up to 40 nuclei with bi- or tri-nucleated variants. There were no mitoses in giant cells in either case.

In Case 1, there was extensive ulceration and regenerative changes in the surface epithelium. Despite the history of three previous recurrences of low grade noninvasive urothelial carcinoma there was no urothelial neoplasia in the present material. The tumor revealed an infiltrative growth pattern but there was no lymphovascular invasion.

**Figure 3:**
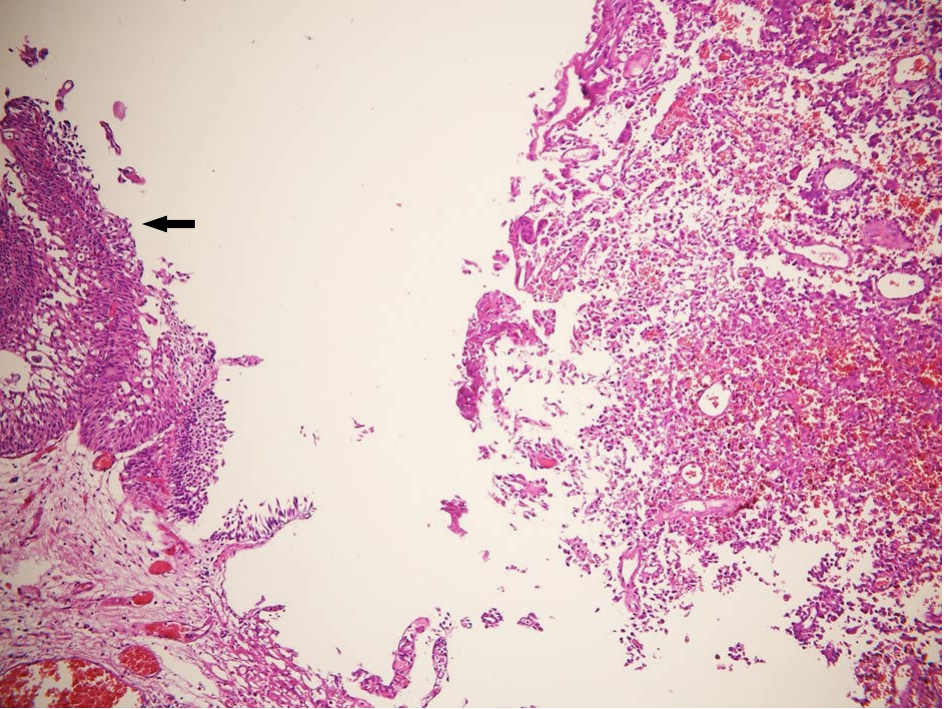
**Low grade urothelial carcinoma (arrow) and giant cell tumor in Case 2. (H&E, × 100)**.

In Case 2, low grade noninvasive urothelial carcinoma was noted in the surface epithelium. GCT was localized beneath the urothelial tumor in the lamina propria and had a nodular infiltrative growth pattern without lymphovascular invasion (Figure 3).

### Immuohistochemical Findings

**Figure 4:**
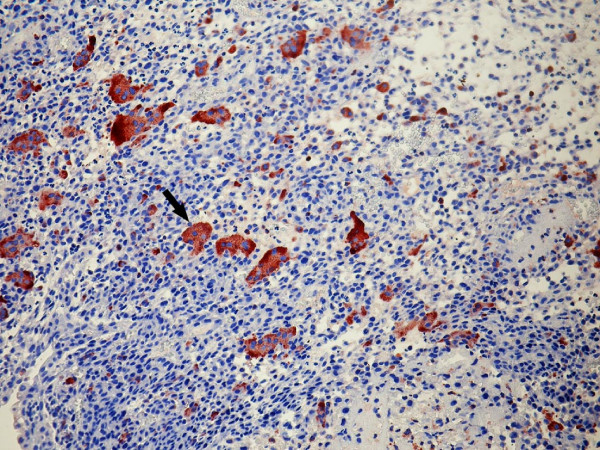
**Giant cells showing cytoplasmic staining with TRAP (arrow) in Case 1**.

Immunohistochemically mononuclear cells in both tumors stained positive with vimentin and CD68. In case 1, there was also focal a-SMA positivity. Ki-67 proliferative index was 5-10% and 2% in the first and second cases, respectively. Less than 10% of mononuclear cells exhibited p53 expression. Giant cells showed vimentin expression, strong cytoplasmic and membranous staining with LCA, and cytoplasmic staining with TRAP (Figure 4) and CD68. Neither mononuclear cells nor giant cells immunohistochemically expressed epithelial markers in both cases.

## Discussion

GCT with its typical biphasic histologic feature including mononuclear cells and osteoclastic cells may be seen in different localizations and described under a variety of names such as giant cell tumor of bone, giant cell tumor of soft tissue, reparative granuloma, giant cell tumor of tendon sheath (local and diffuse), and giant cell tumor in visceral organs.

GCT arising in visceral organs is uncommon and it is extremely rare in urinary tract with only 9 cases in urinary bladder reported in literature to date (Table 2). The terminology and histogenesis of the GCT in visceral organs is still unclear and somewhat controversial. Tumors localized in urinary bladder and pancreas have been named as osteoclast-rich undifferentiated carcinoma in the recently published reports [12,13]. The presence of a synchronous epithelial tumor in most of the cases, as well as immunohistochemical expression of p53 and some epithelial markers in mononuclear cell component, and aggressive clinical behavior were the main reasons for the nomenclature used in various organs.

**Table 2:** Literature Review of Giant Cell Tumor of Urinary Bladder

Authors	Age/sex	Expression of histiosytic markers and vimentin in MC	Expression of epithelial markers in MC	Associated malignancies	Treatment	Outcome
Holtz et al [7]	79/M	NA	NA	UC	TUR	Death at 33 months (unrelated cause)

Kitazawa et al [8]	67/M	NA	NA	UC, noninvasive	TUR	NED (8 months)

Ligdi et al [9]	67/F	NA	Keratin (-) EMA (-)	UC, noninvasive	TUR	NED (18 months)

Amir and Rosermann [10]	74/M	Vimentin (+)	Keratin (-) EMA (-)	UC, noninvasive	NA	NA
	69/M	Vimentin (+)	Keratin (-) EMA (-)	UC, noninvasive	NA	NA

O'Conner et al [11]	73/F	NA	NA	None	TUR, Anterior pelvic	Exenteration+ vaginectomy

Baydar et al [12]	81/M	CD68 (+)	Keratin (-) EMA (-)	UC, noninvasive	TUR	NA
	81/M	CD68 (+)	Keratin (-) EMA (-)	UC, noninvasive	TUR	Alive, recurrent UC (4 months)
	67/M	CD68 (+)	Keratin (-) EMA (+)	UC, noninvasive	Radical cystectomy	Death at 1 year

Behzatoglu et al (New cases)	56/M	CD68 (+) Vimentin (+) SMA (+)	Keratin (-) EMA (-)	None	TUR	NED (35 months)
	74/M	CD68 (+) Vimentin (+)	Keratin (-) EMA (-)	UC, noninvasive	TUR	NED (42 months)

UC, urothelial carcinoma; NED, no evidence of disease; NA, not available; MC, mononuclear cells

Kitazawa et al was the first to describe urothelial carcinoma having the morphology of "giant cell tumor of the bone". Since then nine similar cases have been reported to date. In the single case by Kitazawa et al and the other two cases by Amir and Rosenmann, non-invasive low-grade papillary carcinomas accompanied giant cell tumors without any direct connection between two tumor components or any other additional morphologic findings that may suggest a form of neoplastic transformation. They also concluded that morphological features in these cases were similar to those of giant cell tumor of the bone and/or reparative granuloma. Additionally, as invasion is an unexpected feature in low-grade carcinoma and giant cell tumor in these tumors are apparently located beneath the neoplastic or nonneoplastic epithelium one can easily propose that these tumors (i.e. low-grade carcinoma and giant cell tumor) represent two distinct entities rather than a transformation from one. Holtz et al, in 1972, first employed the term carcinosarcoma in their report of a case similar to that of Kitazawa et al. The patient survived for 33 months and died because of another cause. Baydar et al reported that one of the three such cases died. Only one of the 11 reported patients including our two cases died due to the disease. This is also consistent with the concept that aggressive behavior might be expected in conventional giant-cell tumor. Furthermore giant cell tumor of bone-like morphology is not a usual pattern in anaplastic-pleomorphic carcinoma.

The tumors in our both cases were histopathologically similar to osteoclastic giant cell tumor of bone and soft tissue. They were composed of round, oval and occasionally spindle shaped mononuclear cells and osteoclastic giant cells. Mononuclear cells in both tumors displayed minimal pleomorphism. Few mitoses (1-2/10 HPF) were noted in Case 1 which had an extensive surface ulceration. Osteoclastic cells were primarily seen within the area of oval to round mononuclear cells rather than spindle mononuclear cells. Moreover, these cells had a tendency to concentrate around areas with extravasated erythrocytes. Tumors also had a rich vasculature. Despite the history of relapsing urothelial carcinoma in Case 1 there was a nontumoral surface epithelium with no foci of an urothelial tumor. On the other hand, noninvasive low grade urothelial carcinoma was seen in Case 2. Immunohistochemically, mononuclear cells expressed vimentin and histiocytic marker CD68. Giant cells expressed vimentin, TRAP, LCA and CD 68 pointing out a mesenchymal lineage. The exact nature of both osseous and extraosseous osteoclastic giant cell tumors is still controversial although many cells of origin -including undifferentiated mesenchymal, monohistiocytic, endothelial, reticuloendothelial and epithelial- have been suggested for extraosseous GCT. One of the most important and consistent features of GCT in urinary bladder is its frequent association with an epithelial neoplasia. In addition, immunohistochemical expression of p53 and some epithelial markers in GCT is also noteworthy. These features led some authors such as Baydar *et al *to describe these tumors as 'osteoclast-rich undifferentiated carcinoma' [12]. Similar terms including 'undifferentiated carcinoma with osteoclast-like cells' and 'osteoclast-type giant cell carcinoma' were also used for tumors in pancreas and salivary glands, respectively [13,14].

Nearly half of the extraosseous giant cell tumors express at least one or two epithelial markers immunohistochemically. Additionally, mononuclear cells in most of these tumors also express histiocytic markers and vimentin, similar to 'de novo' GCT not accompanied by epithelial tumors [4,15,16]. Therefore, it may be assumed that morphological features of mononuclear cells in GCT as well as features suggesting a histiocytic lineage are similar in both soft tissue and visceral tumors. Osteoblastic differentiation is prominent in bone whereas synovial features are more pronounced in tendons [17,18]. In any circumstances, all GCTs have a common morphology and low degree of aggressiveness. In addition, p53 expression can be seen not only in GCT of visceral organs but also of bone, and the malignant form of GCT of bone has been presented as a new entity in the recent WHO classification [19-21].

The term 'undifferentiated carcinoma' should be reserved for the tumors mainly composed anaplastic carcinoma cells (spindle cells, pleomorphic giant cells or bizarre cells). Osteoclasts may also be seen in carcinomas of visceral organs, gastrointestinal stromal tumors and leiomyosarcomas [22-24]. These tumors should be clearly differentiated from tumors with typical morphological features of GCT accompanying to an in situ or invasive carcinoma to prevent an overdiagnosis of 'undifferentiated carcinoma'.

The prognosis of pure 'de novo' GCT in visceral organs is similar to their bone counterparts and aggressive behavior is very rare, thus total excision is an adequate treatment choice. Immunohistochemical expression of keratins is a common feature both in 'de novo' tumors and in tumors accompanying an epithelial tumor. GCT is expected to be seen 'de novo' in visceral organs similar to other mesenchymal tumors located in organs other than bone and soft tissue. However, simultaneous presence of epithelial tumor whether benign or malignant is still a subject of controversy causing a diagnostic challenge. A common carcinogen affecting both epithelial and stromal cells may be a possible explanation.

We believe that tumors in urinary bladder exhibiting morphologic features similar to GCT of bone should be evaluated separately from the accompanying epithelial tumors, if any, and should be designated as 'giant cell tumor' due to their favorable prognosis.

## Competing interests

The authors declare that they have no competing interests.

## Authors' contributions

KB and HD performed microscopic evaluation, conducted the design of the study and drafted the manuscript. ÖA and ŞC participated in histological evaluation and the design of the study. GEH and MÖ participated in immunohistochemical evaluation and helped to draft the manuscript. GÖ and PY conceived of the study, and participated in its design and coordination and helped to draft the manuscript. All authors read and approved the final manuscript.

## Consent

Written informed consent was obtained for publication of this case report and accompanying images. A copy of the written consent is available for review by the Editor-in-Chief of this journal.
